# Topics of Public Interest

**Published:** 1913-02

**Authors:** 


### TOPICS OF PUBLIC INTEREST.

---

Telegraphic Reports of Communicable Diseases to the Public Health Service.—At the Tenth Annual Conference of State and Territorial Health Authorities with the United States Public Health Service, held in Washington, June 1, 1912, a resolution was submitted by the conference committee on Morbidity Reports and adopted by the conference, providing for the telegraphic report (collect) to the Surgeon General of the Public Health Service of the occurrence of cases of certain diseases of special importance; also telegraphic reports of outbreaks and epidemics of certain diseases, the telegrams in each instance to be followed by a report by mail. Provision is also made for monthly reports of notifiable diseases and for special reports of outbreaks of small pox in which fatal cases occur.

Blanks have been prepared for:

(1) The reports to be mailed subsequent to the telegraphic report of the occurrence of cholera, typhus fever, yellow fever, plague and Rocky Mountain spotted (or tick) fever.

(2) The regular monthly reports.

(3) Reports of outbreaks of smallpox with fatal cases.

---

Commission to Investigate Vivisection.—January 14, 1913, State of New York. No. 153. In Senate. Introduced by Senator McClelland, read once and referred to the Judiciary Committee. An Act to Create a Commission to Investigate the Present Condition and Extent of the Practice of Experimentation on Living Animals in This State and to Report what Changes, if Any, in the Existing Laws, are Desirable to Protect Animals from Unnecessary Suffering in this Practice without Unreasonably Interfering with Legitimate Scientific Research.

The People of the State of New York, represented in Senate and Assembly, do enact as follows:

Section 1.—The Governor is hereby empowered to appoint a commission which shall consist of five (5) members, two (2) of whom shall be physicians or persons experienced in the practice of vivisection and residing within this State, two (2) of whom shall be active members of some organization within this State having for its purposes the prevention of cruelty in Animal Experimentation, and the remaining member of which commission shall be appointed by the Governor at-large.

Sec. 2.—Such commission shall investigate and report the present condition and extent of the practice of experimentation on living animals in this State and the amount of avoidable cruelty involved therein; also make a full inquiry into the present condition of the law of this State for the protection or regulation of scientific investigation or research of this character; also consider the condition and effectiveness of the law for the prevention of abuse in animal experimentation or of unnecessary crulty to animals therein. It shall inquire what further legislation, if any, may be needed to prevent unnecessary suffering of animals through such practice or its abuse, without interfering with properly conducted and legitimate scientific experiments by competent experts. For these purposes the said commission is hereby authorized to investigate the practice as it exists, to send for persons or papers to administer oaths and to examine witnesses and papers respecting all matters pertaining to this subject. This commission shall serve without compensation and shall make a full and final report to the Governor within one (1) year after its appointment, including such recommendations for legislation as in its judgment may seem proper.

Sec. 3.—This act shall take effect immediately.

Published by the Society for the Prevention of Abuse in Animal Experimentation, 204 Montague street, Brooklyn, N. Y. This society expressly disavows sympathy with anti-vivisectionists and the medical profession is largely represented on its advisory board. The officers are: President, J. B. Y. Warner, Rochester, N. Y.; Vice-President, Jefferson Seligman, New York; Treasurer and Counselor, Frederick P. Bellamy; Secretary, Mrs. William Vanamee, 204 Montague St., Brooklyn.

This bill should receive careful and impartial consideration by the medical profession, unless it can be shown—as does not seem likely—that there is some sinister motive behind the bill, it should receive professional support. Otherwise it is not improbable that the medical profession will be accused of concealing abuses and popular opinion will support the demand of anti-vivisectionists for a drastic law that will seriously impede scientific and humanitarian advancement.

---

MORTALITY IN HUNTING.—Twenty-seven hunters and 15,000 deer were killed in New England during the season just ended.

---

A COMPULSORY VACCINATION BILL has recently been killed in the Vermont legislature.

---

DENTAL INSPECTOR FOR NIAGARA FALLS.—The Board of Education has asked for an appropriation of $1,000 for this object.

---

INFANTILE PARALYSIS.—A case was reported in Niagara Falls, December 31, the first in several months.

---

FOUR CHILDREN IN ONE YEAR.—On January 13, 1912, Mrs. John A. Klemm of Des Moines gave birth to twin girls, still alive and well; and on January 3, 1913, to twin boys, each weighing seven pounds and vigorous.

---

MERRY CHRISTMAS.—A St. Louis doctor's wife presented him with a razor strop made from the skin of a leg which he amputated and which she preserved and had tanned. If Santa Claus has brought any of our readers a gall stone watch charm or any similar cheerful reminder of professional prowess and wifely devotion, we shall be pleased to record the fact.

---

ACTING DENTAL SURGEONS.—Examinations will be held at Fort Slocum, N. Y., Columbus Barracks, Ohio, and elsewhere, April 7. Appointments are for three years at $150 per month, with promotion on further examination at the end of the period, to the grade of dental surgeon with relative rank of first lieutenant. Application should be made to the Surgeon General, U. S. Army, Washington, D. C. There is a large number of vacancies.

---

CHIEF OF DEPARTMENT OF MEDICINE, PHILIPPINE GENERAL HOSPITAL.—This position will be filled on evidence of fitness as indicated by application and examination submitted in writing. The salary is $4,000. Applicants must be under 40. Apply for Form B. 1, A. 2, to U. S. Civil Service Commission, Washington, D. C.

THE IOLA SANITARIUM (Monroe County Tuberculosis Hospital), makes an interesting showing in its annual report. Beginning with 10 patients in October, 1910, it has gradually increased to an average daily attendance of 91, about evenly divided among incipient, moderately advanced and advanced cases, of all ages. Of 242 discharges, 62 were by permission, 7 were references to Raybrook, Saranac and return to old country, 3 were expelled, 41 left against advice, 52 absconded and 84 died. Obviously with no power to retain cases against their will, and with the admission of all grades of the disease, a high proportion of cures is not to be expected, and very conservative and non-committal claims are made in this regard:

| | Apparently Recovered. | Arrested. | Improved. | Unimproved. |
|---|---|---|---|---|
| 62 Incipient....... | 10 | 12 | 14 | 25 |
| 48 Mod. Advanced. | 1 | 6 | 19 | 22 |
| 48 Advanced...... | 0 | 1 | 24 | 23 |

pupils, a total of 105 especially dangerous to others; 30
Of 272 persons 31 were engaged in handling foods, making clothing, etc.; 37 in house work and 37 were school laborers and 10 teamsters were included, a noteworthy fact in reference to etiology, as the occupations imply out-door work. On the other hand, indoor labor did not exceed 5 to an occupation (shoemakers) and was usually 1 or 2 to an occupation. Here is food for thought.

---

MORAL IMBECILITY.—One of a gang of burglars recently caught in Buffalo was deliberately trephined, a few years ago, in the hope of improving his mentality and moral appreciation. While the operation evidently did not produce absolute success, it introduces a peculiar medico-legal element into the prosecution, and we venture to predict that the man cannot be imprisoned except in a suitable institution as a defective.

---

PHYSICIAN GIVES SITE FOR CAMPUS.—Dr. John P. Munn, New York City, president of the United States Life Insurance Company, has offered the University of Rochester a lot 370 by 170 feet at the corner of Prince street and University avenue as a site for the Women's Campus.

---

THE NAVY LEAGUE OF THE UNITED STATES seeks legislation to reorganize the personnel of the navy, to favor early promotion, and to obviate too early retirement of fleet commanders; also to determine a consistent policy of naval construction by the establishment of a Council of National Defense. We are asked to support a petition to Congress along these lines. It seems unwise to give this support because a medical editor is presumably, and in the present instance, actually, lacking in expert knowledge on such a question. We may say, however, that on general principles, rapid promotion early in a career, and retention of active service for a long term after securing a high position, seems to imply a large organization, and one rapidly increasing, whether we deal with the navy, the army, or anything else. Without in any way favoring a niggardly or short-sighted policy, the present problems of this country are largely economic and demand retrenchment to the fullest degree compatible with safety.

---

MEASLES assumed the degree of an epidemic in North Tonawanda on New Year's Day. The school at La Salle and several in Niagara Falls have been closed.

---

ELECTION BOOTH USED TO HOUSE CONSUMPTIVE IN NIAGARA FALLS.—Owing to the failure to provide a county institution, a legless man will be thus cared for. While it would seem that, with proper precautions, tuberculous patients might be kept in general hospitals indefinitely, in the absence of special institutions, the State law is explicit in this respect. Old street cars have been suggested as almost ideal bungalows for consumptives —though it seems that they might prove drafty and afford niduses of infection—and an election booth might also be modified so as to make a fairly good hospital for one or two patients, in an emergency.

---

THE SALARY OF THE STEUBEN COUNTY BACTERIOLOGIST has been fixed at $1,500 by the supervisors, $500 to be appropriated from the treasury if the fees are not adequate.

---

MARRIED NURSES.—So much has been said in criticism of the marriage of nurses that the statistics of the Children's Hospital of Buffalo (Twentieth Annual Report, October, 1912), may be of interest. Out of a total of 78 graduate nurses from 1903 to 1912, 1 has died and 12 are married. Considering that nurses are of the usual marriagable age and selected and trained so as to represent a class superior to the average, this does not seem to show an excessive rate.

---

WASTE OF SEWAGE.—Professor King of the University of Wisconsin estimates the annual waste, per 1,000,000 population at 5 to 12 million pounds of organic nitrogen; 2 to 4 million pounds of potassium; 1 to 3 million pounds of phosphorus. A comparison is made with the thrift of the Chinese who have supported their dense population with no loss of fertility of the soil.

DISTRIBUTION OF WEALTH.—Charles B. Spahr in 1895 published the following statistics. We cannot vouch for their accuracy and would be glad of information on the subject:

| Class. | No. of Families. | Percent. of pop. | Ave. wealth per family | Aggregate wealth. | Percent. total wealth |
|---|---|---|---|---|---|
| Rich ....... | 125,000 | 1.0 | 263.040 | 32,880,000,000 | 54.6 |
| Middle .... | 1,362,500 | 10.9 | 14,180 | 19,320,000,000 | 32.2 |
| Poor ...... | 4,762,500 | 38.1 | 1,639 | 7,800,000,000 | 13.0 |
| Very poor.. | 6,200 000 | 50.0 | ...... | ............. | .... |
| Total .... | 12,500,000 | 100.00 | 4,800 | 60,000,000,000 | 100.0 |

---

THE NEW YORK STATE BOARD OF REGENTS has barred the graduates of 22 medical colleges of the United States and Canada from practicing in the State, unless they shall comply with the State Board's requirements. The institutions are as follows: Oakland College of Medicine and Surgery, Oakland, Cal.; Medical Department, Howard University, Washington, D. C.; Chicago College of Medicine and Surgery, Chicago, Ill.; Valparaiso University Medical Department, Valparaiso, Ind.; Hahnemann Medical College of Chicago; College of Physicians and Surgeons, Chicago; College of Homeopathic Medicine, State University of Iowa; College of Medicine, State University of Iowa; Kansas Medical College, Department of Washburn College, Topeka, Kan.; College of Physicians and Surgeons, Baltimore; School of Medicine, Boston University; Medical Department, Creighton University, Omaha; Dartmouth Medical School, Hanover, N. H.; Cleveland-Pulte Medical College, Cleveland, O.; College of Medicine, University of Tennessee, Memphis, Tenn.; Medical Department, Vanderbilt University, Nashville, Tenn.; Milwaukee Medical College, Department of Marquette University, Milwaukee, Wis.; Wisconsin College of Physicians and Surgeons, Carroll College, Milwaukee, Wis.; Manitoba Medical College, Winnipeg; Faculty of Medicine, Dalhousie University, Halifax, N. S.; Faculty of Medicine, University of Toronto, Canada; Faculty of Medicine, McGill University, Montreal, Quebec.

---

REPORT OF BUREAU OF DEPORTATION, STATE OF NEW YORK.

| | 1912. | 1911. | Per Ct. of Inc. |
|---|---|---|---|
| Number new cases under observation....... | 2,704 | 1,934 | 39.8 |
| Deported by U. S. Immigration Service.... | 419 | 345 | 21.4 |
| Repatriated at expense of state............ | 474 | 204 | 132.8 |
| Repatriated at expense of friends...... | 278 | 235 | 18.3 |
| Total .............................. | 1,171 | 784 | |

| | | | |
|---|---|---|---|
| Non-residents returned to other states: | | | |
| Expense of state | 295 | 151 | 95.3 |
| Expense of friends | 287 | 191 | 50.4 |
| Total | 582 | 342 | |
| Total aliens deported and non-residents returned | 1,753 | 1,126 | 55.7 |

PROPORTION OF IMMIGRATION DESTINED TO NEW YORK, 1905-12.

| Year. | Total immigration. | No. destined to N. Y. State. | Per cent. destined to N.Y. State. |
|---|---|---|---|
| 1905 | 1,026,499 | 315,511 | 31.0 |
| 1906 | 1,100,735 | 374,708 | 34.0 |
| 1907 | 1,285,349 | 386,244 | 30.0 |
| 1908 | 782,270 | 256,425 | 32.7 |
| 1909 | 751,786 | 220,865 | 29.4 |
| 1910 | 1,041,570 | 280,880 | 26.9 |
| 1911 | 878,587 | 258,113 | 29.4 |
| 1912 | 838,172 | 239,275 | 28.5 |
| Total | 7,704,968 | 2,332,021 | .... |
| Annual average | 963,121 | 291,503 | 30.3 |

RATING OF MEDICAL SCHOOLS.—The Council of Medical Education (Jour. A. M. A., January 18, 1913), has made an interesting report. We give only the statistics:

Class A Plus. 4-year schools, 22 (including P. & S., Cornell, University and Bellevue and Syracuse for N. Y.) 2-year schools, 2.

Class A (acceptable but lacking in certain details); 4-year schools, 31 (including N. Y. Homoeop., Albany and Buffalo, for N. Y.) (2 to be merged). 2-year schools, 6.

Class B (needing general improvements); 4-year schools, 22 (one will be converted into a post-graduate school in three years, one will be discontinued and one gives only last two clinical years); 2-year schools, 1.

Class C (requiring complete reorganization); 4-year schools, 27 (6 not recognized as in good standing by their state boards, 2 about to be reorganized).

Class A for colored students, 2.

Class C for colored students, 2.

Total schools conferring degrees, 106, three to be discontinued. Net total schools conferring degrees, 103.

While we have long advocated improvement in medical education and, for economic reasons, restriction of the number of schools, there are some considerations that should tend to a charitable attitude. For example, there are quite a number of physicians of ten to forty or more years standing who have done quite creditable work. Yet not more than a very few obtained their diplomas on courses that would now be graded even in Class C.

We are conducting a practice and a journal as well as possible under existing circumstances, but we should be very much surprised to have either marked A plus—and for the same reason that some very excellent medical schools are graded only A or B, lack of money. It is the present fashion to cast stones at medical institutions. Let us all forego this agreeable exercise until we are sure we are not, ourselves, living in green houses. It is perfectly possible that a school may fulfill a real need and be honestly and conscientiously conducted and yet not be successful in obtaining a large supply of tainted money.

It is not unreasonable to interpret the sixth commandment as applying to human activities as well as human existence.

On the other hand, the merging of weak schools into strong ones is to be commended. The Birmingham Medical College, rated as B, has carried out an idea that we have had for some time: After graduating the three classes already in attendance, it will be converted into a post-graduate school. There are a number of institutions, fairly well equipped but not actually needed to augment the already excessive numbers of the profession, and not financially able to comply with the ever increasing standards of undergraduate education. As centers of professional club life, libraries, museums and laboratories for investigation, these schools would fulfill a higher mission and there would be no objection to conceding to their present faculties all the prestige and opportunities for emolument that they enjoy at present.

---

Fifth Year of Medical Study will be required in Pennsylvania after 1914. The additional year will, however, amount practically to compulsory interne service, of which 90 per cent. of graduates already avail themselves.

---

Deaths and Injuries Due to Surface, Elevated and Suburban Traction Cars. In New York in 1912, 290 persons were killed; 30,000 passengers and 7000 employes were injured. 1378 injuries were serious.

N. A. R. D. PRESCRIPTION PRICING SCHEDULE (all fees are compounding fees only.)

1.—*Liquid Prescriptions*—Minimum charge, 25c. All sample or compound mixtures, internal or external, dry or liquid, eye or private disease remedies, and veterinary preparations are included in this table.

| Quantity. | Dose. 1-5 M. | Dose. 10-25 M. | Dose. ʒss-ʒi | Dose. ʒii-ʒi | Gargle and External only |
|---|---|---|---|---|---|
| ½ oz........ | .25 | .20 | .15 | .10 | .10 |
| 1 oz........ | .35 | .30 | .20 | .15 | .10 |
| 2 oz........ | .45 | .35 | .25 | .20 | .15 |
| 3 oz........ | ... | .40 | .30 | .25 | .20 |
| 4 oz........ | ... | ... | .35 | .30 | .25 |
| 6 oz........ | ... | ... | .40 | .35 | .30 |
| 8 oz........ | ... | ... | .45 | .40 | .35 |
| 12 oz........ | ... | ... | .55 | .45 | .40 |
| 16 oz........ | ... | ... | .60 | .55 | .50 |
| 32 oz........ | ... | ... | .75. | .70 | .65 |

2.—*Proprietaries*—Original package, regular retail price. When costing over $2, $4 or $8 per dozen, add 65 per cent. to cost. When transferred to new container, add 15 per cent. to regular retail price. When part of package is dispensed, double cost of amount used and add charge for container (see below).

3.—*Dry Mixtures*—Minimum charge, 15c. These figures are compounding fees only. Pills, Powders, Capsules, Wafers, etc.

| Number ....... | 4 | 6 | 8 | 10 | 12 | 15 | 20 | 24 | 30 | 40 | 50 |
|---|---|---|---|---|---|---|---|---|---|---|---|
| Fee (revised)... | 15c | 20c | 25c | 30c | 35c | 40c | 45c | 50c | 60c | 75c | 90c |

Then every additional 25 up to one hundred, 25c. After that 20c for every additional 25.

Where powders are prescribed by the ounce, charge as follows for compounding fee: 1 oz., 25c; 2 oz., 35c; 3 oz., 40c; 4 oz., 45c; 6 oz., 50c; 8 oz., 55c; 12 oz., 65c; 16 oz., 75c, etc.

Proprietaries costing 20c per hundred or less, 10c for labeling and package, and 15c for 1 doz., 25c for 2 doz., 35c for 3 doz., 40c for 4 doz., then 5c for each additional dozen. Costing over 20c and under 50c per hundred, 10c for labeling and package, and 20c for 1 doz., 35c for 2 doz., 50c for 3 doz., 60c for 4 doz., then 10c for each additional dozen. When the wholesale price is over 50c per hundred, special rates may be made.

4.—*Fatty Mixtures, etc.*—Ointments and Cerates.

| | | | |
|---|---|---|---|
| ½ oz.... .20 | 2 oz.... .35 | 4 oz.... .55 | 8 oz.... .75 |
| 1 oz.... .25 | 3 oz.... .45 | 6 oz.... .65 | 16 oz.... 1.00 |

Suppositories, Bougies, etc.

| | | | |
|---|---|---|---|
| 1....... .20 | 5....... .45 | 12....... .75 | 24....... 1.35 |
| 2....... .30 | 6....... .50 | 15....... .90 | 30....... 1.60 |
| 3....... .35 | 8....... .60 | 18.......1.05 | 36....... 1.80 |
| 4....... .40 | 10....... .65 | 21.......1.20 | |

5.—*Veterinary.*—Allow a discount of 25% from the regular schedule on compounding fee only, except that for bulk powders the minimum charge be 25c for compounding.

6—*Household Remedies, Mixtures, etc.*—Add regular retail price of ingredients (none less than 5c) and charge for container. If any compounding is necessary, charge at rate of $1.50 per hour.

7—*Containers*—Pill and Powder Boxes, 5c. Ointment Boxes, 5c. Ointment Jars, 1 oz., 5c; 2-4 oz., 10c; 8 oz., 15c. Bottles, 8 oz., or less, 5c; 10-16 oz., 10c; 32 oz., 15c; ½ gal., 20c; 1 gal., 25c. Glass Stoppered Bottles, three times the price of plain bottles.

8—*Marking Price of Prescriptions.*—If a prescription or copy leaves your store, mark it with N. A. R. D. price, as follows:

| | | | | | | | | | |
|---|---|---|---|---|---|---|---|---|---|
| P | H | A | R | M | O | C | I | S | T |
| 1 | 2 | 3 | 4 | 5 | 6 | 7 | 8 | 9 | 0 |

9—*Admissable Changes.*—If customer is poor, add a star (*) to price mark, showing you have gone below Schedule Price. If your present prices are lower than above, raise them gradually to Schedule Prices.

10—*How to Fix Price. This is Important.*—The price of the Prescription is the Compounding Fee, plus the cost of the container, plus twice the cost of the material. (Exception: When the cost of material is over $1.00, multiply cost by 1½ instead of 2; and further, if the cost is over 50c and under $1.00, adopt the following sliding scale: Cost 60c, add $1.10; cost 70c, add $1.20; cost 80c, add $1.30; cost 90c, add $1.40. These prices are based on an approximate $1.50 per hour scale.

*Example.*—If the ingredients of a four-ounce mixture cost 12c, compute price as follows: Compounding fee, 35c plus container, 5c plus twice cost of material, 25c (12x2)—total, 65c.

---

Grain Receipts at Buffalo.—Mr. Junius S. Smith, Lake Weighmaster of the Buffalo Corn Exchange, reports for 1912 the receipts of 108 million bushels of wheat and of other grains up to nearly 160 millions, total. On the average estimate of a barrel of flour or five bushels of wheat per capita per annum, the elevators of Buffalo handle the cereal food of approximately 25 million persons.

---

Giantess Dead.—Emma Ewing died last month in Gorin, Mo., aged 30. She was of usual stature till her ninth year. Her height was 8 feet 4 inches.

